# The Influence of College Entrepreneurship Education System on the Cultivation of Applied Innovative Talents

**DOI:** 10.3389/fpsyg.2022.844234

**Published:** 2022-05-10

**Authors:** Xiaoqi Zhao, Xiaorong Wang

**Affiliations:** College of Basic Medicine, Hebei North University, Zhangjiakou, China

**Keywords:** educational concept of higher institutions, innovation and entrepreneurship, cultivation of applied innovative talents, entrepreneurship education, entrepreneurial talents

## Abstract

With China’s socioeconomic development, especially in the educational sector, foreign advanced education experience might not be omnipotent for the innovation and entrepreneurship education (IEE) in Chinese colleges. In response to such a difficult context, firstly, this study was conducted to analyze the applied innovative talent cultivation status quo in Chinese colleges under innovation and entrepreneurship. Then, the authors dug into the current situation and the development of IEE-related courses in College S through Questionnaire Survey (QS) and unveiled the efficacy of entrepreneurship environment and conditions, IEE courses, teaching methods, and policy system on the college applied innovative talents cultivation. Finally, the experiment discovered that three problems are prominent when training applied innovative talents in College S: unreasonable talent training process, imperfect education system, and emphasizing theory over practice. The main reason is that the IEE courses are not systematically set up, and teachers are in severely short supply. According to the research outcomes, the corresponding countermeasures and suggestions were proposed for applied innovative talents cultivation in College S. It is concluded that the cultivation of applied innovative talents under innovation and entrepreneurship should be reformed from four aspects: educational concept, educational model, educational policy, and social support, laying a foundation for improving the quality of IEE in Chinese colleges and universities and strengthening the cultivation of applied innovative talents.

## Introduction

It has been argued that the cultivation of innovative talents in colleges should fully be combined with their own educational resources under the guidance of new educational concepts, and there is a need to connect the talent cultivation objectives with the enterprise social demands (Zilkha, 2018). In this way, students can obtain a higher quality and knowledge structure within a certain cultivation time to meet social development needs (Shkabarina et al., 2020). As the main base for cultivating innovative talents, colleges’ educational concepts and methods determine the quality of the talents. As trainees, college students should join the innovation and entrepreneurship team and cultivate themselves into the applied innovative talents in the new era (Zheng and Liu, 2020). Colleges’ broad teaching platforms and rich teaching resources (Sadiyev, 2021) are requisite for colleges to cultivate applied innovative talents. However, the analysis of the current situation and employment of applied talents cultivation in major domestic colleges has uncovered the deficiencies in the current cultivation process. The employment of college graduates is still extremely challenging (Mets et al., 2017; Lin and Lin, 2021).

In 2010, China’s Ministry of Education pointed out that colleges’ education should be based on the concept of promoting innovation and entrepreneurship education (IEE) and the goal of improving the quality of talent cultivation. However, a colleges’ education is different from technology and cannot fully learn from foreign advanced experience. It is necessary to comprehensively consider factors such as China’s national conditions, the development status of domestic enterprises, and the education level of major colleges and universities. Through a series of investigations, the current problems of IEE in Chinese colleges and universities and the factors that affect college students’ innovation and entrepreneurship behavior are clarified, and targeted countermeasures are put forward. On this basis, it is possible to formulate an IEE system that conforms to the actual situation of Chinese colleges and universities by putting forward hypotheses and exploring the impact mechanism of various factors on college students’ innovation and entrepreneurship. Therefore, it is the key to clarify the current situation of IEE in Chinese colleges and universities and analyze its existing problems. Based on this, it firstly analyzes the current situation of the cultivation of applied innovative talents in Chinese colleges and universities under the background of innovation and entrepreneurship. Then, taking the college students of College S in China as the research object, the current situation of IEE in College S and the development of education courses related to innovation and entrepreneurship are investigated by means of a questionnaire. Finally, it analyzes the influence of entrepreneurship environment and conditions, innovation and entrepreneurship courses, teaching methods and policy systems on the cultivation of applied innovative talents in colleges and universities. The innovation of this paper is that it comprehensively considers China’s national conditions, enterprise development, and university education level and makes an in-depth investigation on the cultivation of innovative and entrepreneurial talents under the new educational concept. It provides an important reference for improving the quality of IEE in colleges and strengthening the training of applied innovative talents.

## Literature Review

“Innovation” is integral to advancing the development of the society as well as improving the quality of life. Papakostas et al. (2021b) pointed out that innovative technology has always been an important part of firefighting as it improves the safety and effectiveness of firefighters (Papakostas et al., 2021b). Troussas et al. (2021) explored the importance of innovative technology, Social Networking for Advancing Knowledge in E-learning environment (SNAKE), in students’ acquiring knowledge and improving their performance (Troussas et al., 2021). Krouska et al. (2021) pointed out that most students prefer to use their mobile devices such as smartphones or Ipad. The integration of games into the educational process may increase students’ motivation and improve their learning outcomes. Research has found that innovative learning models based on mobile games have a significant positive impact on the engagement and academic performance of students (Krouska et al., 2021). Papakostas et al. (2021a) pointed out that the innovative behavior of integrating augmented reality (AR) technology into welding training can largely improve the efficiency, safety and time gain of the operation, and reduce the cost of consumables and infrastructure (Papakostas et al., 2021a). At present, some scholars have carried out a series of work on the reform of IEE system in colleges and universities. Gianiodis and Meek (2020) integrated logic in stakeholder theory to provide a framework for explaining the relationship between entrepreneurship education and the formal and informal processes of technology commercialization within entrepreneurial universities. In addition, a series of questions and educational evaluation indicators were developed to assess the entrepreneurship education system within entrepreneurial universities, reflecting the needs of a wider range of stakeholders, including administrators, students and the office of technology commercialization (Gianiodis and Meek, 2020). Jena (2020) firstly investigated the cognitive, affective, and behavioral components of entrepreneurship education among Indian college students. Secondly, it explored the influence mechanism of students’ attitude of entrepreneurship education on entrepreneurial intention. Finally, the role of control variables (such as gender and entrepreneurial family background) in the relationship between attitudes of entrepreneurship education and entrepreneurial intentions was investigated. The results showed that attitude toward entrepreneurship education has a significant positive impact on entrepreneurial intention (Jena, 2020). Iwu et al. (2021) collected information on students at a university in South Africa and explored factors that may influence students’ entrepreneurial intentions. Empirical results showed that the respondent group strongly agrees with the usefulness of entrepreneurship education for economic development, indicating that they were well aware of the role and benefits of entrepreneurship at the macro level. The study also found that the perceived ability of the lecturer team was moderately positively correlated with the students’ entrepreneurial intention. This shows that colleges and universities must take certain responsibilities to ensure that the teachers of relevant entrepreneurship education courses are highly competent and can motivate students to form entrepreneurial intentions (Iwu et al., 2021). To evaluate the effectiveness of entrepreneurship education of university in Tehran, Sherkat and Chenari (2022) studied the effects of entrepreneurship courses, entrepreneurship education and university climate on the effect of students’ entrepreneurial intention, implementation intention and commitment level by using entrepreneurial intention as an indicator of the effectiveness of entrepreneurship education The findings show a positive correlation between entrepreneurship education and entrepreneurial intentions (Sherkat and Chenari, 2022). Boldureanu et al. (2020) explored the mechanism of exposure to successful entrepreneurial models on students’ entrepreneurial intentions. The findings demonstrate that entrepreneurship education based on successful entrepreneurial models may positively influence students’ entrepreneurial attitudes and intentions. It also emphasizes that if educators want to improve the efficiency of education that focuses on building entrepreneurial skills, they should design different courses for business and non-business students (Boldureanu et al., 2020).

The above research results manifest that the IEE carried out by colleges and universities has a positive correlation with the generation of college students’ entrepreneurial intentions. A reasonable education system is conducive to the cultivation of applied innovative talents. Meanwhile, the setting of innovation and entrepreneurship courses in colleges and universities should be formulated according to the actual situation of schools and students, rather than blindly applying existing achievements. However, at present, most Chinese colleges and universities do not pay attention to the improvement of the IEE system, and still draw on the advanced experience of other countries, which may no longer meet the actual needs. Therefore, it is an urgent problem to investigate and analyze the current situation of IEE in China’s major universities, and formulate a more reasonable education system according to the actual situation.

## Research Methodology

### Overview of Related Concepts and Theories

#### Overview of IEE

**Figure 1:**
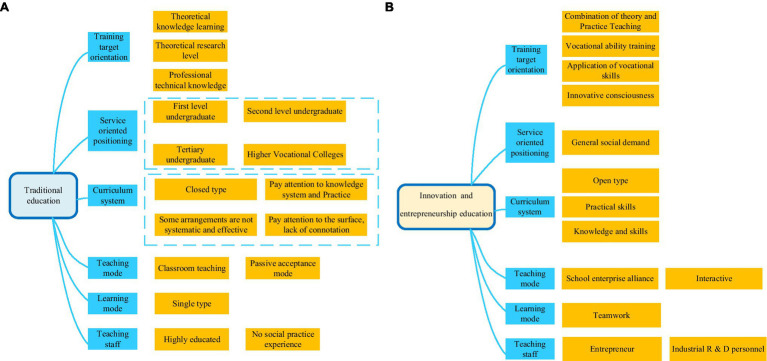
Comparative chart of traditional education and innovation and entrepreneurship education (IEE) in colleges. **(A)** Traditional education; **(B)** IEE.

IEE includes higher education (undergraduate education and graduate education), as well as secondary vocational education and higher vocational education. Currently, most domestic colleges cultivate research and academic talents. The new model of higher education reform is to cultivate compound talents (Rosa et al., 2018). In this context, colleges must change the traditional educational concepts and methods, reform the curriculum structure, and change the classroom structure. Meanwhile, there is a need to change the knowledge acceptance and transfer model between students and teachers (Wu and Song, 2019; Qi and Wang, 2020). Figure 1 shows the comparison between traditional education and IEE in colleges.

#### Competency-Based Education

**Figure 2:**
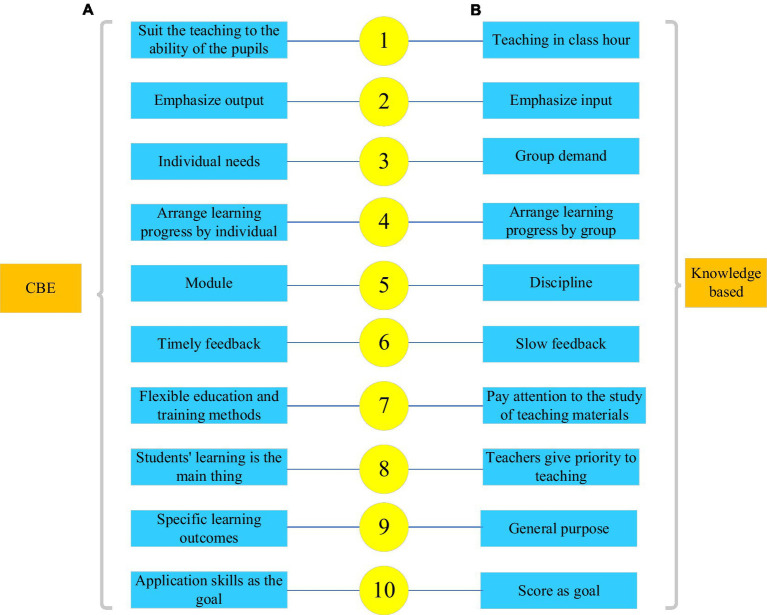
Competency-based education (CBE) versus knowledge-based education (KBE) **(A)** CBE; **(B)** KBE.

According to competency-based education (CBE), top industrial talents who had a clear view of the current situation and development of the industry were recruited by colleges, thereby forming a competency-based committee of experts who can analyze industrial or job requirements. Then, talents were divided through hierarchical and individual-level solutions to evaluate their characters and quality, based on which a judgment on their competency for a specific industry is judged so that more clear cultivation objectives were scheduled. Further, colleges organized teaching staff to develop corresponding courses that appealed to each cultivation ability and objective, and appropriate teaching contents were formulated. After the teaching tasks and cultivation plans were completed, the cultivated talents were assessed (Wu and Wu, 2019). The comparison between CBE and traditional knowledge-based education (KBE) in China is shown in Figure 2.

Figure 2 shows that the core of CBE is the cultivation of students’ competency. Compared with KBE, CBE can better adapt to the social development needs, which is more in line with the cultivation of applied talents under the new educational concept of colleges (Wu W. et al., 2019).

#### Talent Classification and Higher Education Classification Theory

**Figure 3:**
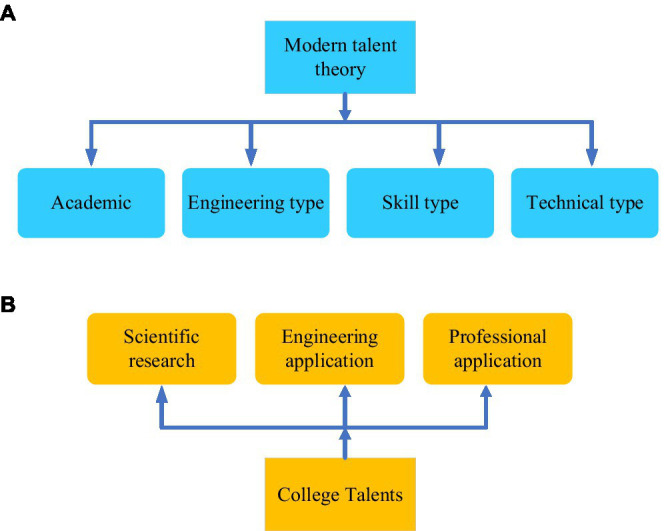
Talents classification **(A)** Modern talent theory and **(B)** college Talents.

Modern talent classification theory and domestic academic higher education talent classification are shown in Figure 3.

According to Figure 3, from the perspective of talent theory classification and university talent classification, higher vocational and technical education should be given proper attention as a kind of higher education (Wu Y. C. J. et al., 2019). In promoting IEE, applied innovative talents from higher vocational and technical education are also in high demand. At the same time, higher education should deepen the classification of vocational application and cultivate corresponding talents based on CBE.

### Current Situation of Applied Innovative Talent Cultivation

IEE has been gradually integrated into the education strategy of colleges that actively carry out IEE, adopted a series of practical exploration, and achieved certain results. The practical exploration mainly includes three aspects (Radovi et al., 2021).

#### Establishment of Innovation and Entrepreneurship Academy

In recent years, with the policy requirements of the MoE and the international education system reform, numerous colleges have established the innovation and entrepreneurship academies to cultivate applied innovative talents adaptable to the trend of times. These academies have achieved some success in recent years, so the cultivation of applied innovative talents has been comprehensively promoted (Reiter et al., 2020).

#### Implementation of Innovation and Entrepreneurship Competitions

In 2015, the MoE held the first *“Internet +” Innovation and Entrepreneurship Competition for College Students*, which has promoted the enthusiasm of college students to participate in innovation and entrepreneurship and is actively responded by colleges that have advanced the practice of new educational concepts (Rizvi et al., 2019). Various entrepreneurship competitions have been held at provincial and university levels, which have become the base for colleges to implement mass entrepreneurship and innovation education to encourage and guide students’ entrepreneurship (Wu et al., 2020). Ideally, competitions can deepen innovation of entrepreneurship education by actively inspiring and guiding students to focus on the needs of the society, thereby promoting the combination of industry, universities, scientific research, and application. The core idea of entrepreneurship competitions is to integrate innovation entrepreneurship education into talent cultivation.

#### Reform of Basic Courses of Innovation and Entrepreneurship

The traditional education classroom is the teacher’s lecture, the students passively accept the knowledge, and their initiative for learning is not high, which is impossible to stimulate the students’ innovative ability. Gradually, colleges are reforming the traditional education model toward student-centered innovative entrepreneurial classrooms by swapping the roles of students and teachings, resulting in an energetic classroom atmosphere for innovation and entrepreneurship, and the enthusiasm for innovation of college students has been aroused by enriching teaching methods and improving teaching plans. Additionally, some colleges have developed compulsory courses and elective courses for IEE and incorporated them into credit management (Savitskyi and Parshakov, 2020).

### The Applied Innovative Talent Cultivation Process

The cultivation process of applied innovative talents is mainly divided into three dimensions.

#### Guiding Students to Set Clear Goals

College students are believed to be the largest national talent pool, and their values, ideal, and faith take shapes during their college years, so the dominating role of schools should be given full play during higher education to help students to acquire knowledge and to establish correct values, ideals, and faith. Meanwhile, schools need to encourage college students to set up life goals agreeing with social demands and self-development (Siringi, 2019).

#### Applied Innovative Talent Cultivation

The cultivation of applied innovative talents should follow talents’ growth, and each individual is independent and unique. Hence, innovative talent cultivation must adhere to the rules of developing talent growth. Particularly, the applied innovative talents and traditional skills talents are distinct groups, and the applied innovative talents should be cultivated from three aspects. Firstly, applied innovative talents need to acquire rich knowledge theories, complete knowledge structure, and clear, logical thinking ability. Secondly, knowledge alone is not enough. They need to combine knowledge with practice and acquire good industrial skills or techniques based on academic knowledge. Thirdly, an overall perspective is a must, including the independent problem-solving ability, thinking, and judging, as well as strong execution and team spirit (Barba and Atienza, 2018; Bar and Baskan, 2020).

#### Applied Innovative Talent Cultivation Method

**Figure 4:**
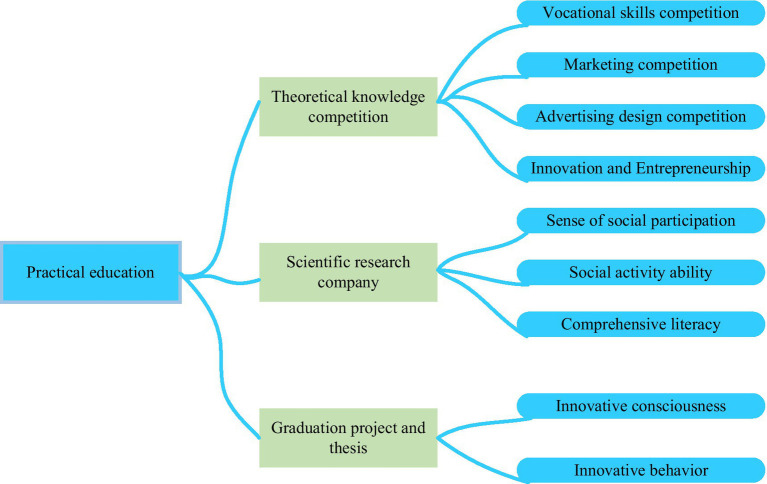
Applied innovative talent cultivation.

Colleges vigorously reform the new curriculum and the knowledge system and promote the students’ innovation ability by setting up related innovative entrepreneurial classes. However, the role of the new teaching model has not yet been fully exerted, students’ autonomous learning and creative practice of understanding are not deep enough, and time and space for the students’ innovative thinking and innovative practice are very limited. Therefore, applied innovative talents’ cultivation mainly consists of three aspects, as shown in Figure 4.

### QS of IEE in College S

A Questionnaire Survey (QS) was conducted on College S. The QS contents include College S students’ understanding of entrepreneurship environment, types and expectations of IEE, scientific research situation of College S, innovation and entrepreneurship policies, and enterprises’ evaluation of talents.

**Figure 5:**
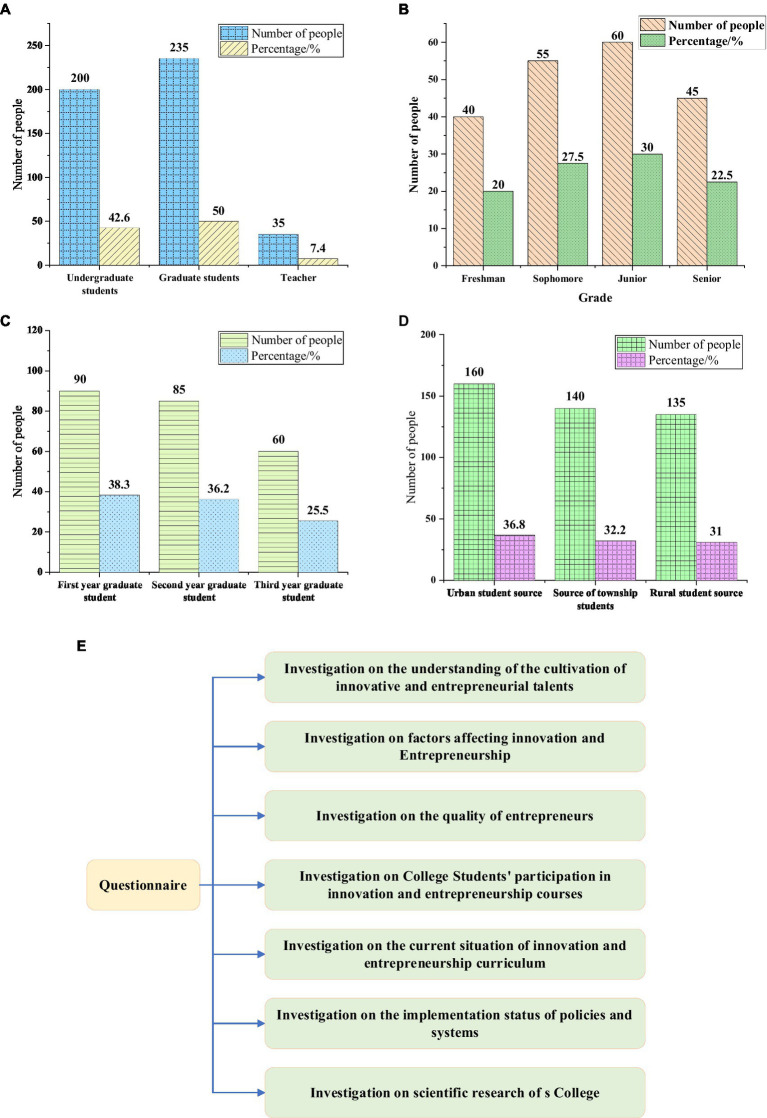
Information of respondents. **(A)** Distribution of undergraduates, graduate students, and teachers. **(B)** Distribution of undergraduate students in different grades. **(C)** Distribution of graduate students in different grades. **(D)** Distribution of students in different locations of their home. **(E)** Content structure of the questionnaire.

Totally, 500 QSs were distributed based on the QS star platform, and 470 QS were returned, with an effective rate of 94%. The respondents of the QS were students and teachers of all grades in College S. The relevant information of the respondents and the content structure of the questionnaire are shown in Figure 5.

As shown in Figure 5, among the respondents of this survey, the proportion of undergraduates is 42.6%, the proportion of graduate students is 50%, and the proportion of teachers is 7.4%. The proportions of freshman to senior students are 20%, 27.5%, 30%, and 22.5%. The proportions of students from cities, towns, and rural areas are 36.8%, 32.2%, and 31%, respectively. The questionnaire mainly involves seven aspects of investigation, namely, the investigation of college students’ understanding of cultivation of innovative and entrepreneurial talents, the investigation of factors affecting college students’ innovation and entrepreneurship, the investigation of the qualities required by entrepreneurs, the investigation of college students’ participation in innovation and entrepreneurship courses, the investigation of the current situation of innovation and entrepreneurship courses, the investigation of the implementation of relevant policies and systems, and the investigation of the current situation of scientific research in College S. The way to obtain the results of the questionnaire survey is as follows: the valid questionnaires are sorted and encoded into the Statistical Product and Service Solutions (SPSS) 26.0 software, and archived. Data analysis are performed using SPSS and Analysis of Moment Structure (AMOS) 19.0 software.

## Results and Discussion

### Analysis of QS Results

**Figure 6:**
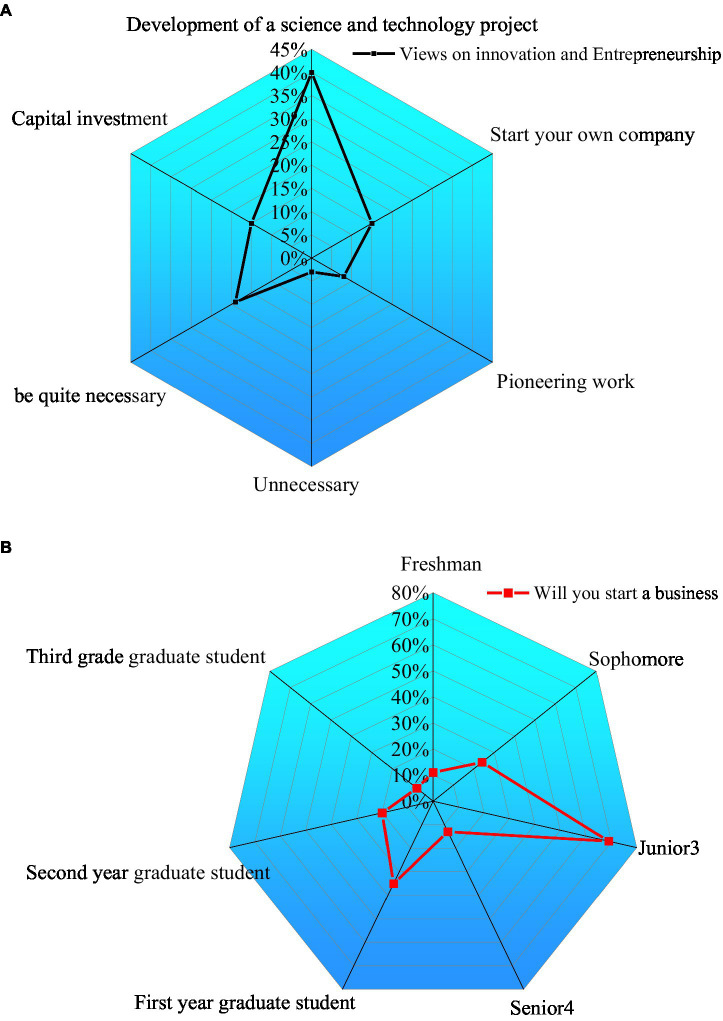
College students’ understanding of the cultivation of innovative and entrepreneurial talents. **(A)** How do you view innovation and entrepreneurship? **(B)** Are you planning to start a business.

1. The QS results of college students’ understanding of the cultivation of innovative and entrepreneurial talents are shown in Figure 6.

As in Figure 6A, 15% of college students believe that innovation and entrepreneurship are “starting their own company,” and 40% of college students believe that it is “developing a new technology or project.” 8% of college students understand innovation and entrepreneurship as “pioneering work.” On the other hand, 15% of college students understand innovation and entrepreneurship as a kind of capital investment, and 3% of students think IEE is not necessary at all. The above results corroborate that the importance of IEE and promoting college students’ employment has not been highly valued in Chinese higher education. Therefore, although IEE courses are offered in colleges, there are still many deficiencies in the specific publicity and implementation process. To this end, it is still necessary to further strengthen the publicity and promotion of IEE to make more students realize the importance of IEE in developing their careers and recognizing their value.

As in Figure 6B, 60% of junior students and 35% think they will start a business. Thus, these two groups have high entrepreneurial enthusiasm. Junior students have more spare time and fewer scientific research tasks. Therefore, they have a strong desire to start a business. Besides, junior students face the choice of postgraduate entrance examination and work and need to improve their self-quality in all aspects to gradually expand the planning and assumption of future career and life.

**Figure 7:**
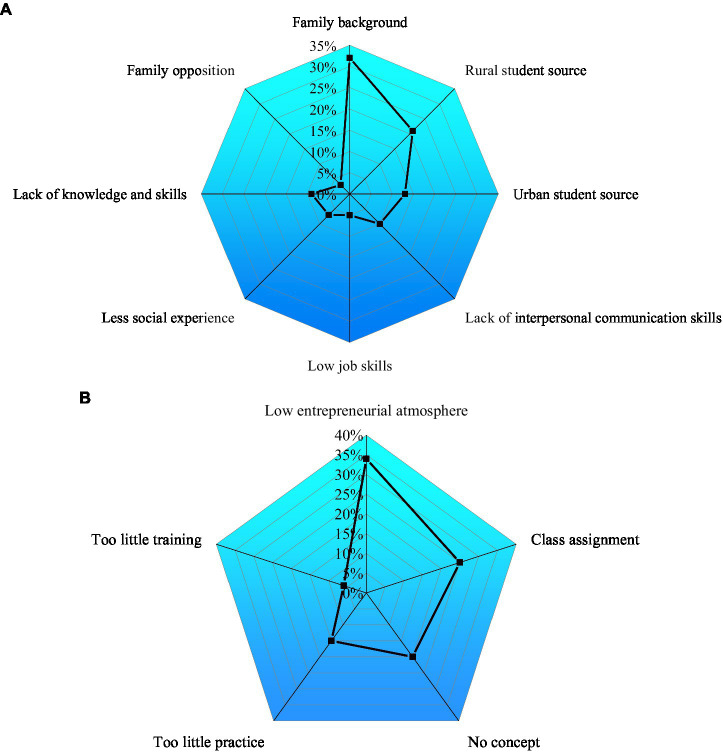
Factors influencing college students’ innovation and entrepreneurship. **(A)** Innovation and entrepreneurship and **(B)** teachers’ views.

2. The factors influencing college students’ innovation and entrepreneurship were analyzed, as shown in Figure 7.

Figure 7 illustrates that family background is the major factor of college students’ innovation and entrepreneurship, and urban students have a more innovative and venturous tendency over rural students, which is believed to be caused by their different educational backgrounds. Meanwhile, interpersonal skills, work skills, social experience, knowledge skills, and family members’ attitudes will have an impact on college students’ innovation and entrepreneurship. In Figure 7B, 35% of teachers believe that the innovation and entrepreneurship courses of colleges do not have a satisfactory effect because the courses do not improve the innovation atmosphere; 25% of the teachers think that “class separation” will college students’ attendance in innovation and entrepreneurship courses, 20% of the teachers think that students are not clear about the development trend of innovation and entrepreneurship. 5% and 13% of the teachers thought that schools have not carried out enough cultivation or practice activities on innovation and entrepreneurship. Apparently, the innovation and entrepreneurship of college students show a general trend of lacking an entrepreneurial atmosphere. Therefore, it is urgent to reform the existing IEE.

**Figure 8:**
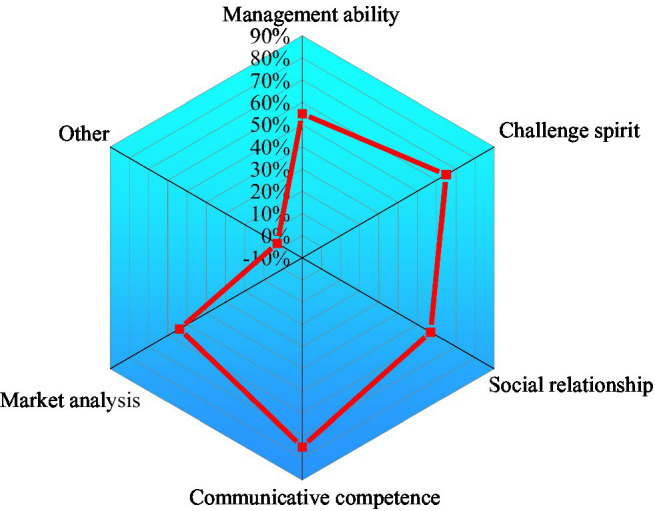
Innovation and entrepreneurship quality of college students.

3. Innovation and entrepreneurship quality of college students are shown in Figure 8.

Figure 8 displays that innovative entrepreneurs’ self-factors are principal. For example, in the basic quality of innovative entrepreneurs, communicative competence accounts for 72.24%, challenge spirit accounts for 66.55%, and social relationship accounts for 57.52%. College students think that communicative competence and challenging spirit are the most important for college students to innovate and start their own business. Besides, practice bases, teaching faculty, and innovation and entrepreneurship cultivation are also important.

**Figure 9:**
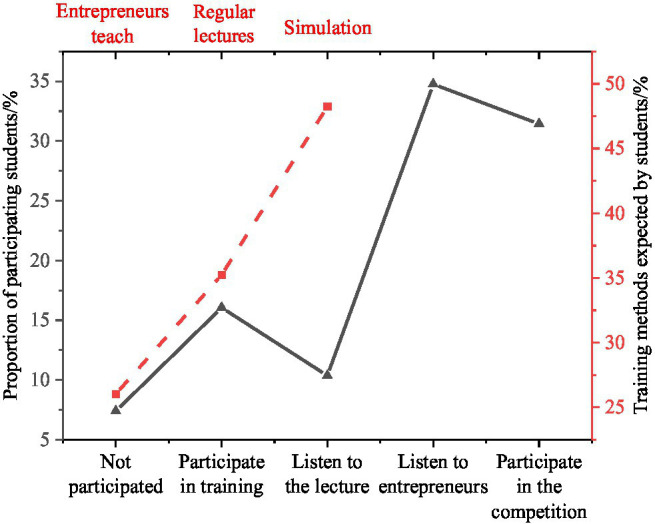
College students’ participation in innovation and entrepreneurship courses.

4. In the teaching process, students participated in IEE, and the results are shown in Figure 9.

Figure 9 shows that listening to the entrepreneurs’ lectures (31.44%) and participating in the competitions (34.76%) are college students’ central innovative undertakings. By comparison, 7.39% of college students are not involved in entrepreneurship education mainly because the school innovation and entrepreneurship courses are not reasonable. This leads to students’ lack of innovation, so their concept of motivation is low.

**Figure 10:**
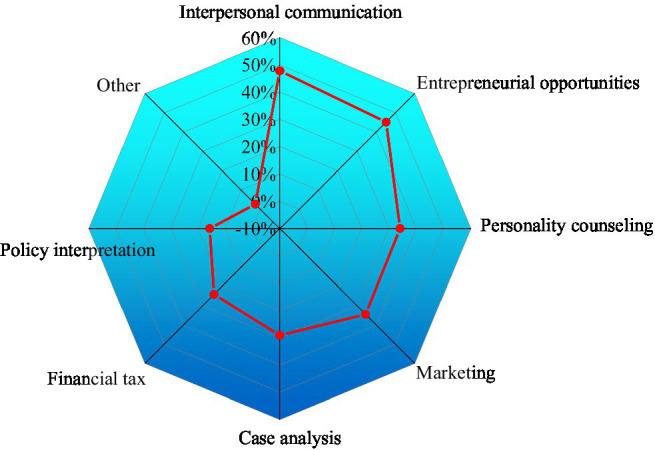
Innovation and entrepreneurship teaching methods.

5. The teaching methods of innovation and entrepreneurship are shown in Figure 10.

Figure 10 displays that college students’ most favorable entrepreneurship courses should include reasonable curriculum setting and cultivation goals, as well as interpersonal communication courses, accounting for 47.82% and 45.15%, respectively. This proves that the current innovation and entrepreneurship courses in colleges emphasize theory rather than practice. The development needs of the entire market and the internal needs of students are not fully considered in the entrepreneurship course design.

**Figure 11:**
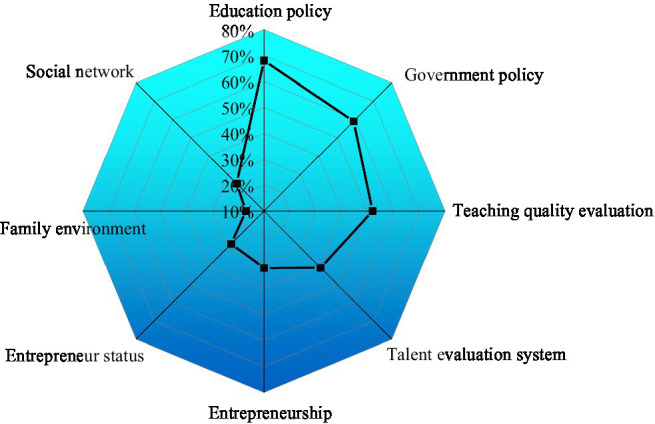
The influence of policies and systems on innovation and entrepreneurship.

6. The policy environment and system are perfect, and the results are shown in Figure 11.

As in Figure 11, education policy, government policy, teaching quality evaluation, and talent evaluation system significantly impact the cultivation of applied innovative talents, with a relative influence of 68%, 59%, 52%, and 41%, respectively. Apparently, educational policies have the most significant impact on the cultivation of applied innovative talents, followed by government policies. In contrast, the family environment has the most negligible impact on cultivating applied innovative talents. The above results show that improving policy environment and system is the most fundamental guarantee for the smooth development of innovation and entrepreneurship. In optimizing the talent training mode of colleges, there is a need to pay more attention to the improvement of relevant policies and systems.

**Figure 12:**
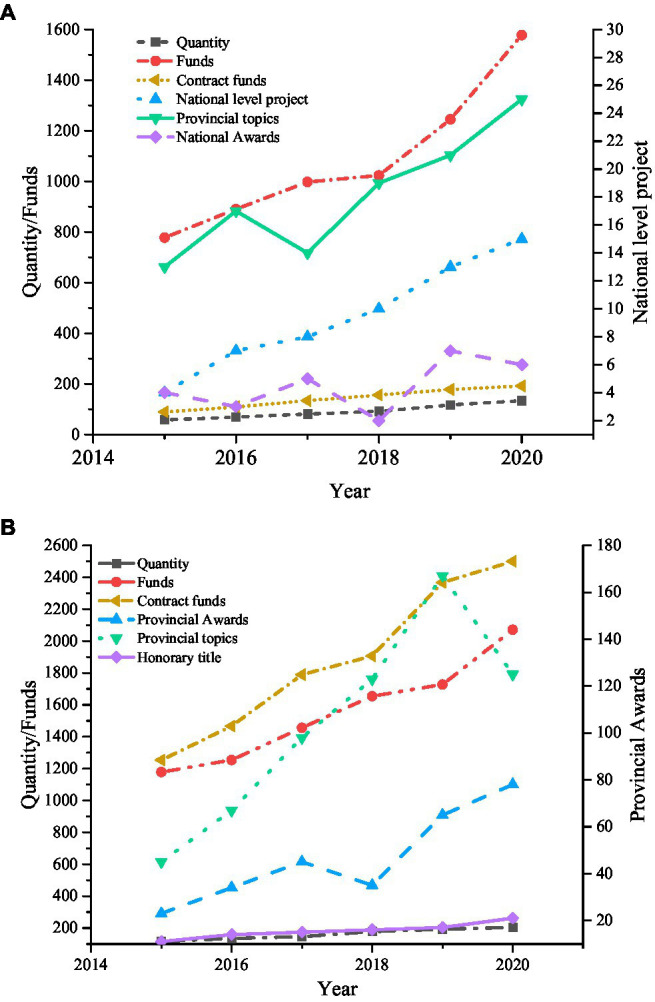
Research funds and projects. **(A)** Projects in science and engineering and **(B)** horizontal projects.

7. The scientific research situation of College S is shown in Figure 12.

**Figure 13:**
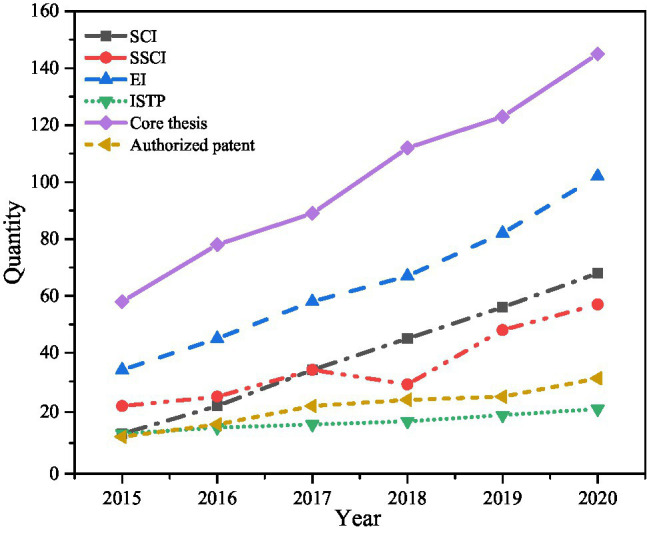
Statistics of scientific research achievements.

Figure 12 reveals that College S has conducted more horizontal projects, spent more project funds, obtained more rewards and titles over science and engineering. Meanwhile, the trend of nearly 6 years shows that College S has developed well in both projects. Next, College S scientific research was statistically investigated from 2015 to 2020, as shown in Figure 13.

Figure 13 implies that the scientific research achievements of College S in recent years have been rich and increasing year by year, which is closely related to the reform of IEE. The College S has strengthened cooperation with enterprises, has sufficient research projects and funds, and achieved more scientific research achievements.

### Analysis on the Cultivation of Applied Innovative Talents in College S

The process of cultivating applied innovative talents.

#### Fuzzy Positioning

**Figure 14:**
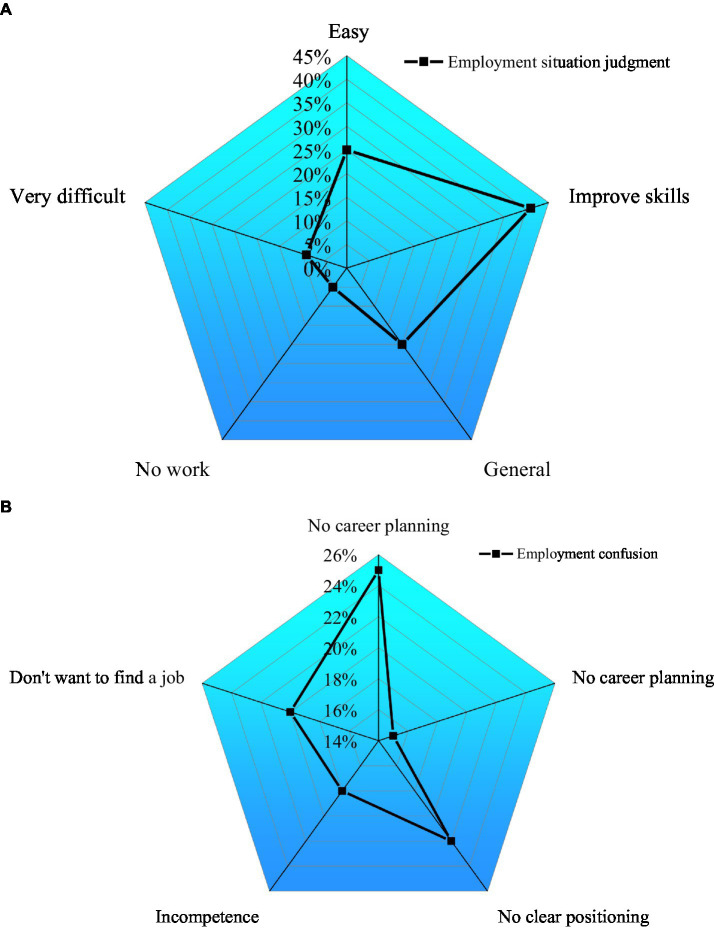
Research funds and projects. **(A)** Evaluation of the situation of innovation and entrepreneurship and **(B)** employment confusion.

As a result of the inaccurate cultivation objectives of applied innovative talents, the positioning of talent cultivation was relatively fuzzy, as shown in Figure 14.

Figure 14A illustrates that fuzzy talent cultivation orientations are common in College S. Therefore, even though many professional students are cultivated, they still lack a sense of direction and clarity in their career development, which will eventually harm their practical work abilities. Figure 14B demonstrates that students with unclear career planning account for 25% of the total respondents, and 25% of the respondents do not know what jobs to choose, showing that students are confused in innovation, entrepreneurship, and employment.

#### Inaccurate Positioning

Schools pay much attention to the cultivation of job skills while ignoring the continuous cultivation. The reason is that colleges have no accurate orientation for talent cultivation. This leads to difficulty cultivating compound talents with strong social ability, high professional quality, and strong post-working ability.

The system problem of applied innovation talent cultivation

1. The curriculum system is not scientific

**Figure 15:**
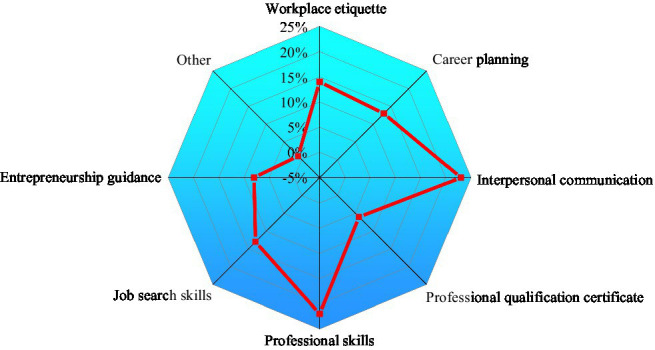
The courses needed for the cultivation of applied innovative talents.

The current curriculum of College S is displayed in Figure 15.

As in Figure 15, the current curriculum structure of College S focuses on professional skills training and interpersonal communication, followed by workplace etiquette. However, College S has not paid enough attention to career planning and entrepreneurship guidance, which also play an essential role in cultivating applied innovative talents. Therefore, the curriculum of College S is not very reasonable, and the teaching contents of IEE courses need to be improved according to the actual situation and needs of students.

**Figure 16:**
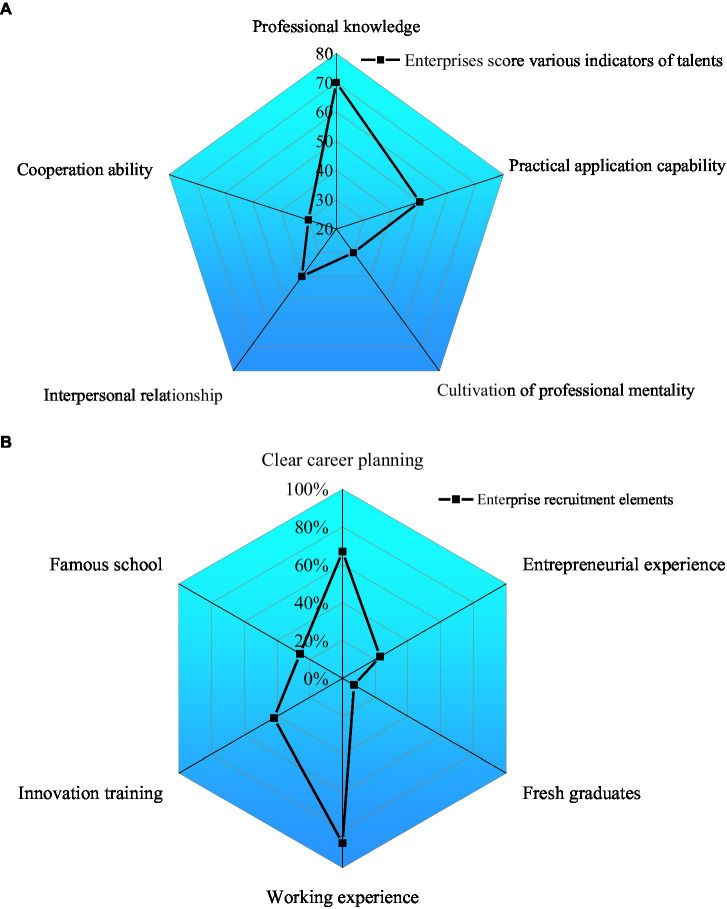
Practical problems of talent cultivation. **(A)** Enterprise talent evaluation indexes and **(B)** factors of enterprise recruitment.

2. The practical problems of talent cultivation are ignored, and the results are shown in Figure 16.

As in Figure 16A, professional knowledge scores the highest (70) among the enterprise talent evaluation indexes. The practical application ability is 50. The score of professional mentalities is lower than 50. Evidently, the students of College S have a good grasp of theoretical knowledge, but their practical operation ability is weak. Meanwhile, there is a problem of emphasizing theory over the practice in college curricula. As in Figure 16B, enterprises prioritize employees with particular work experience, clear career planning and high comprehensive quality. According to these findings, schools may adjust and optimize the corresponding curriculum content to align with the actual needs.

### The Reasons for the Problems in the Cultivation of Applied Innovative Talents in College S

Colleges’ curriculum of mass entrepreneurship and innovation is not systematic and lacks teaching resources. Moreover, College S is faced with stereotyped education method and unsound curriculum system, yet it has not recognized the importance of IEE to the cultivation of applied innovative talents. Besides, the mass entrepreneurship and innovation education is only set as an elective course in College S, leading to inadequate initiative and enthusiasm for innovation and entrepreneurship.

### Strategies for Applied Innovative Talents Cultivation Under the Background of Innovation and Entrepreneurship

**Figure 17:**
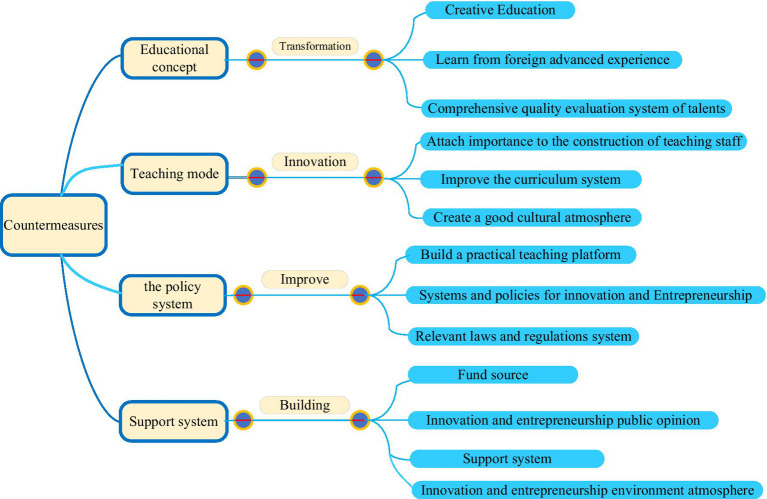
Strategies for cultivating applied innovative talents in College S.

Given the shortcomings in applying innovation talent cultivation in College S, the following countermeasures were put forward from four aspects, as shown in Figure 17.

Figure 17 presents that the countermeasures are mainly divided into four aspects: the change of educational concept, the innovation of teaching mode, the improvement of policy systems, and the establishment of social support system. Corresponding suggestions and countermeasures are put forward for these four aspects.

## Discussion

Research on the cultivation of innovative and entrepreneurial talents is earlier in other counties (Moberg et al., 2020). In some countries, such as the United States, the cultivation of innovative talents began as early as 1940. Although there are different definitions of talent cultivation, the methods and concepts of talent cultivation adopted are not significantly different from those in China (Glatter, 2017). In the 1940s, American colleges and universities opened Master of Business Administration (MBA) courses for IEE, and then discussed innovative education models. By allowing teachers to go into the enterprise and understand the latest development and needs of the enterprise, so that students can be exposed to the latest developments in the industry or enterprise, to add some professional courses to train students in a targeted manner (Bowers and Chet, 2017). In some European countries, they have gradually formed their own education systems based on their own national conditions and referenced educational experiences from other countries (Bilak, 2020). In the previous literature review, it has been clarified whether the IEE system in colleges and universities is perfect and reasonable. It has an important influence on the formation of students’ entrepreneurial intentions, and also plays a key role in the cultivation of applied innovative talents. However, at present, most of the IEE systems in Chinese colleges and universities still draw on foreign advanced experience, and it is urgent to formulate an education system that is in line with China’s national conditions. Therefore, it mainly takes the teachers and students of College S in China as the research object, and investigates and analyzes the current status of IEE in the college, the development of relevant courses, the status of policy implementation, and the scientific research of the college. This lays a good foundation for the reform of the IEE system in colleges and universities of China. The research results indicate that a large proportion of students do not have a good understanding of the cultivation of innovative and entrepreneurial talents, indicating that the importance of IEE in colleges’ education and the promotion of college students’ employment has not been highly valued. 35% of teachers believe that the current innovation and entrepreneurship courses offered by schools have no good effect and the course atmosphere is not good enough. 7.39% of college students have not participated in entrepreneurship education. The main reason is that the IEE courses offered by schools are unreasonable, resulting in students lacking the concept of innovation and low enthusiasm. The IEE courses currently provided by the school focus on theory rather than practice. The development needs of the entire market and the inherent needs of students are not fully considered in the design of entrepreneurship education courses.

To sum up, the current IEE system of College S has problems such as ambiguous positioning, unreasonable curriculum setting, and neglect of practice of talent training. The main reason for the analysis is that the IEE courses set up by College S are not systematic, the teacher resources are seriously lacking. It not only has a single educational method, but also has an imperfect curriculum system. In the process of running the school, College S did not realize the importance of IEE to the cultivation of applied innovative talents. It treats entrepreneurship and innovation education only as an elective course, resulting in a lack of enthusiasm and enthusiasm for students to innovate and start a business. Therefore, College S should reform its IEE model from four aspects: educational concept, educational model, educational policy and social support.

## Conclusion

College IEE and talent training cannot mechanically emulate the advanced experience, but it should integrate various factors for targeted research. Accordingly, the present work comprehensively considers factors such as China’s national conditions, domestic enterprises’ development, and colleges’ education level. In this study, an in-depth research was carried out on the cultivation of applied innovative talents. Firstly, the current situation of the cultivation for applied innovative talents in colleges under innovation and entrepreneurship was analyzed. Then, the current situation and development of IEE courses in College S were investigated utilizing QS and the impact of entrepreneurship environment and conditions were examined on IEE courses, teaching methods, and policy systems on the cultivation of applied innovative talents in colleges. The results corroborate that College S has many problems training applied innovative talents due to the unsystematic IEE courses and the severe teaching faculty shortage.

In response to the issues, the corresponding countermeasures and suggestions were provided for the cultivation of applied innovative talents in College S based on the concept of IEE. It is concluded that the cultivation of applied innovative talents under the background of innovation and entrepreneurship should be reformed from four aspects: educational concept, educational model, educational policy, and social support.

The shortcomings of this paper are summarized as follows. The scope of the experiment is limited to the students in College S, and the research results are not representative. Therefore, it is necessary to further expand the range of research in the later stage, continuously improve the research findings, and provide an essential reference for improving the quality of IEE in Chinese colleges and strengthening the training of applied innovative talents.

## Data Availability Statement

The raw data supporting the conclusions of this article will be made available by the authors, without undue reservation.

## Ethics Statement

The studies involving human participants were reviewed and approved by the Hebei North University Ethics Committee. The patients/participants provided their written informed consent to participate in this study. Written informed consent was obtained from the individual(s) for the publication of any potentially identifiable images or data included in this article.

## Author Contributions

All authors listed have made a substantial, direct, and intellectual contribution to the work and approved it for publication.

## Funding

This work was supported by the Social Science Foundation of Hebei Province Exploration and practice of training applied medical talents based on the resources of experimental teaching center (HB17JY007) and the Hebei Province Higher Education Teaching Reform Research and Practice Project (no. 2018GJJG319).

## Conflict of Interest

The authors declare that the research was conducted in the absence of any commercial or financial relationships that could be construed as a potential conflict of interest.

## Publisher’s Note

All claims expressed in this article are solely those of the authors and do not necessarily represent those of their affiliated organizations, or those of the publisher, the editors and the reviewers. Any product that may be evaluated in this article, or claim that may be made by its manufacturer, is not guaranteed or endorsed by the publisher.
